# The impacts of a justice-focused body image program for early adolescents

**DOI:** 10.3389/fpsyt.2026.1828519

**Published:** 2026-06-23

**Authors:** Anna C. Ciao, Kevin Delucio, Alex Park, Lily Ngo

**Affiliations:** Western Washington University, Bellingham, WA, United States

**Keywords:** adolescents, body image, eating disorder, prevention, social justice

## Abstract

To address gaps in universal, diversity-focused eating disorders prevention with early adolescents, our team co-created an evidence-informed body image intervention through a community-engaged, participatory research process. The *Body Justice* intervention and associated research were co-created by a team of middle school students and staff and undergraduate students and faculty in the Pacific Northwest United States. The intervention includes eight brief lessons (six hours total) with culturally-tailored content rooted in cognitive dissonance and media literacy (e.g., cultural appearance ideals, diversity representation within media, food culture). The intervention was delivered with 7th grade students over three years (N = 333; 49% students of color; 53% cisgender boys, 36% cisgender girls, 12% gender diverse; 27% sexually diverse) using college student leaders (near peers) and middle school student co-leaders. Student satisfaction immediately after the intervention was moderate overall and higher for students of color, sexually diverse students, and cisgender girls and gender diverse students compared to their peers with majority identities. Across the sample, there was a significant reduction in unhealthy weight control behaviors from baseline to two-month follow-up with similar improvement among subgroups except for students of color, who had smaller reductions over time compared to their white peers. Across the sample, there was a significant reduction in internalized appearance norms from pre to post-intervention and through follow-up. These reductions were similar across gender, but the change was significant only for white students and straight students. There was no overall improvement in perceived appearance pressure from social media over time, but subgroup analyses revealed that students of color experienced improvement over time unlike other subgroups. In general, subgroup analyses should be interpreted cautiously due to concerns about adequate power. These results suggest that the *Body Justice* curriculum was delivered effectively and was well-liked by middle school students with marginalized identities. While aspects of the intervention were beneficial (e.g., a reduction in unhealthy weight control behaviors over time), findings suggest potentially differential results across identity subgroups. This has implications for collaborative school-based research, body image and eating disorders prevention, and community-engaged methods to foster equity.

## Introduction

As knowledge of eating disorders risk has increased in recent decades, so has our ability to create evidence-based programs to prevent the onset of these illnesses (1). Given the serious nature of eating disorders, the difficulty treating them, and their rising incidence worldwide (2, 3), eating disorder research has prioritized prevention (reducing risk factors; increasing protective factors) and early intervention (identifying and intervening on early symptoms). The most robust evidence base exists within a selective prevention framework, with interventions targeting specific populations at high risk for developing eating disorders, primarily cisgender girls and young women with body dissatisfaction (4). These interventions are successful in reducing eating disorders risk (and in some cases, preventing the onset of eating disorders) using group-based programs rooted cognitive dissonance (5) and other cognitive therapy interventions (6) to target empirically established risk factors (e.g., body dissatisfaction, internalized attitudes about ideal bodies). Given the gender-based risk within eating disorders, this is incredibly promising, and yet it is also important to explore how gender intersects with racial/ethnic identity and other social identities (e.g., sexuality, ability), to identify and determine differential risk across diverse groups. Eating disorders and interventions must be understood in their appropriate sociocultural context in order to identify areas of nuance that may not reflect European and white-dominant norms around body image and body dissatisfaction.

Less attention has been given to universal prevention of eating disorders, where individuals are included in interventions regardless of risk status. While universal prevention (e.g., with all students at a particular grade level in a school) may include those who are *not* currently at elevated risk for developing an eating disorder, this approach can be an important part of the prevention continuum, targeting young people *before* they develop disordered eating and eating disorder symptoms. Universal interventions within the early adolescent developmental period (ages 11-14) may be particularly important, as puberty and sociocultural influences converge to substantially increase risk (7), and research demonstrates that eating disorder symptoms that develop in later adolescence tend to remain stable or get worse over time (8). Furthermore, body image concerns (even those that will not progress to an eating disorder) are associated with poor mental health over time (e.g., low self-esteem, depression, disordered eating; 8), and directly addressing body image among a wide range of adolescents has benefits beyond preventing eating disorders. And yet, body image interventions and eating disorders prevention in early adolescence is underrepresented in the literature, where the majority of studies are with older adolescents and young adults (9–11). In fact, school-based prevention with young people ages 10-15 has been identified as a research priority for the future (12).

While universal, school-based body image programs will include students across the gender and risk spectrum (10), there are surprisingly few programs with an explicit focus on diversity and reducing risk for a diverse range of body image experiences. While existing evidence-based programs are flexible to allow lived experience to be brought into group conversations, programs that were created with the express purpose of including diversity and justice elements are rare, despite body image concerns being widespread across the spectrum of lived experience, including for cisgender boys and men who are often left out of prevention interventions past school age (13).

Recent research also documents the significantly elevated eating disorder risk for individuals with identities that are socially marginalized, including gender diverse individuals, sexually diverse individuals, and racially diverse individuals (14, 15). Body image concerns may be rooted in appearance-related judgements and discrimination that is based on skin color, visible disability, gender expression, and presumptions of sexual orientation (16). We also see health disparities including greater barriers to accessing mental health treatment based on marginalized identity (e.g (17). Universal interventions that include content specifically created to discuss diverse body image experiences have the potential to benefit both individuals who exist in bodies with more privilege and importantly, for those whose bodies are more likely to be oppressed.

Over the last decade, our team has investigated cognitive dissonance-based body image interventions for diverse young adults that use active exercises to encourage young people to directly challenge cultural appearance pressures (18, 19). In recent years, our work shifted to target a younger age range in order to promote body liberation among early adolescents. In 2021, our team began a community-based participatory collaboration with a local middle school to create an evidence-informed and culturally appropriate intervention for early adolescents (20). Two advisory teams (adult and youth) met regularly over the course of an academic year with university faculty and undergraduate psychology students. This team co-created an eight-session curriculum to address body image topics through the lens of the middle school student experience, with intentionality around including young people with diverse, intersecting identities as co-creators and co-writers of the intervention. Although the *Body Justice* lessons were created by and for this specific school context, the intervention exercises are based in media literacy theory (to critically evaluate media sources and content) and cognitive dissonance theory (to practice new ways of thinking that challenge internalized appearance ideals) (9, 10). Both theoretically grounded approaches are noted in **Table 1**, showing a more in-depth look into the program’s content and how specific activities leveraged either or both cognitive dissonance theory and media literacy. Exercises are designed to create a critical awareness of culturally-based appearance norms that are highly gendered and racialized and that privilege bodies representing dominant cultural groups. The current paper describes implementation-effectiveness hybrid research (21). Specifically, we explore effectiveness (impact on disordered eating and body image risk factors) over the first three years of a justice-focused, universal prevention intervention implemented in a new context. Importantly, the size of the sample over time and the racial, gender, and sexual diversity of the students within the school allows for sub-group analyses to explore whether the curriculum is equally beneficial for all students.

**Table 1:** Body justice project session outline and mapping onto intervention theory.

Session	Activity name	Activity description	Cognitive dissonance (How this appears in the activity)	Media literacy (How this appears in the activity)
Week 1 Part 1	Group Norms*	Students generate norms for participating and sharing	N/A	N/A
	*Defining Cultural Ideals*	Students brainstorm physical aspects of the feminine and masculine ideals. Facilitators bring together ideas and emphasize the unrealistic standards for feminine and masculine bodies.	By emphasizing generated traits of each ideal that may contrast other traits of the same ideal (i.e. big breasts but flat stomach), students see that the ideals are unrealistic and logically impossible to adhere to.	By prompting students to think about media portrayals of each ideal (e.g. feminine or masculine), facilitators create a space open for media critique
Week 1 Part 2	*Inclusivity of Ideals*	Students speak in small groups to understand who is left out of the ideals in terms of diverse identities and who is not represented by the media’s portrayal of the ideals.	Through emphasizing who is left out of ideals that are meant for everyone to achieve, facilitators paint how much harder it might be to reach the ideals for those left out of the ideals already.	Through understanding the media's portrayal of the ideals as limited and narrow, students are able to think critically about the representation in their media.
	*The Healthy Ideal*	Students share their ideas about what healthy means. They also share who they have heard these ideas from. Facilitators aid in understanding that the healthy ideal is not one person’s idea but rather externally influenced (i.e. society informs us that to be healthy you have to follow specific rules).	Through the emphasis on being influenced by a restrictive health ideal, students are able to push back and re-envision their own opinions on what healthy looks and feels like.	Through students’ thinking about who tells them what is healthy, facilitators set up an opening to discuss commercialized media as part of the society that pushes healthy ideals onto the students.
Week 2 Part 1	Examining Diet Culture*	Students define diet culture and briefly describe its dominant messages	N/A	N/A
	*Take a Side*	Students will interactively respond to True/False questions by moving their bodies to either side of the room that correlates with an answer (i.e. True or False). Prompts ask students to think critically about different statements about health, body image, and eating behavior; all of which allow for discussion about debunking myths against body compassion/neutrality. Facilitators pause after each statement to hear from both sides of arguments (True or False) before giving in depth explanation of the correct answer.	Students being able to debunk commonly held beliefs in the classroom space allows them to not only feel more agency in explaining themselves, but also informs them of how some commonly held beliefs may not be as true as they once thought. (i.e. Less fat in foods is always good; FALSE, low-fat does not equal healthy)	Students are able to draw from their own sources of information to debate their choices in this activity. This allows them to refer to their sources of information when they are correct, and be critical of their sources when they are incorrect. Either way, students are asked to support their claims in this activity.
Week 2 Part 2	Intuitive Eating*	Students are introduced to the concept of intuitive eating and practice identifying body cues on the hunger-fullness scale.	N/A	N/A
	*Good and Bad Foods*	Students interrogate the pros and cons of placing a moral value on food (e.g., calling a food choice “good” or “bad”)	Students challenge the idea that unhealthy foods are “bad” and healthy ones are “good”.	N/A
	*Favorite Food Memory*	Students write or draw a story of their favorite food memory to elaborate on what food means to them by going past thinking of food as only fuel. Prompts include food being important to culture, connection with others, family, tradition, and more.	By expanding food’s purpose to be more than fuel and connected to a positive memory, students challenge their negative or neutral thoughts towards food and reframe food in positive ways.	N/A
Week 3 Part 1	*Media Representation*	Students generate examples of positive diversity representation within the media, including the personal impact and what they would like to see more.	Students are challenged to name positive examples of diverse bodies in the media, disrupting the idea that media should only depict bodies close to appearance ideals	Students interrogate the presence of diverse body representation within the media they consume on a regular basis and the impact of the media they consume.
	*Analyzing Brands*	Students are given real examples of different brands and their ads for either clothing, food/nutrition, or beauty products. They are asked to answer questions about the ads that result in understanding who is being represented and who is left out; what the pros and cons of the product are; and how inclusive their product may be.	By critiquing brands that lack diverse representation of bodies and interacting with brands that do, students continue reframing their ideas about ideal bodies.	By looking at real examples of ads, the students are able to practice their critical thinking about media they are shown involuntarily/based on their interaction algorithms. This provides the students with hands-on practice of critical media consumption.
Week 3 Part 2	*Social Media*	Students discuss the positives and negatives of social media in their lives and identify ways that social media perpetuates diet culture and appearance ideals.	N/A	Through examples and discussions about filters, algorithms, and influencers, students increase their awareness of how curated their social media experiences can be.
	*What We Say Matters*	Students evaluate and sort common phrases, identifying whether a phrase is appropriate or inappropriate and if it is about appearance or not.	Students become more conscious of commonly used phrases that could be considered positive but are ultimately inappropriate to say to another person (i.e. “You look so skinny in those jeans!”; a positive comment that is about appearance and inappropriate due to the commenting on someone’s appearance in relation to norms outside of our control).	N/A
Week 4 Part 1	*Body Respect*	Students are introduced to the concept of body autonomy and respect and brainstorm ways to engage in self-care toward their body (things that bring their body peace, pleasure, or joy).	Students practice with the idea of caring for their bodies even when they do not feel positive about their appearance.	N/A
	*Body Boundaries*	Students identify personal body boundaries (things they do not enjoy) and generate ideas for responding to negative appearance comments to respect others’ boundaries.	Students practice with setting limits for their bodies and revisit the concept of negative appearance talk to brainstorm ways to challenge comments.	N/A
	*Jeopardy*	Student groups play a jeopardy game that covers all the topics, lessons, and activities from the whole program.	This activity works as a refresher for the past activities.	This activity works as a refresher for the past activities.
Week 4 Part 2	*Sharing the Message*	Students generate a poster to share key ideas from the curriculum	This activity works as a refresher for the past activities.	This activity works as a refresher for the past activities

*Indicates activities that are found in the curriculum but do not rely as heavily on Cognitive Dissonance or Media Literacy Theories.

## Materials and methods

### Participants and implementation timeline

The *Body Justice Project* was piloted with 6th, 7th, and 8th graders. As part of the curriculum planning, collegiate and student leaders collaborated to revise the intervention and have it better reflect student’s experiences. A sustainable implementation plan was co-established where 7th graders would receive the *Body Justice* lessons each year, with program facilitation from trained college leaders and co-leadership from middle school students wherever possible. Additional details on the co-design process are available (20).

*Body Justice* lessons were implemented with 7th grade students for three years in a row (*N* = 333 students). Each year, school leadership (either the assistant principal, principal, or school counselor) reached out to 7th grade teachers to assess interest in bringing the *Body Justice* lessons to their homeroom class. Engagement ranged over the three years of implementation: in 2023, nine 7th grade homerooms participated (n = 145 students) and the curriculum was delivered by 8 college leaders with 12 middle school co-leaders. In 2024, seven 7th grade classrooms participated (n = 113 students), with 11 college leaders and two middle school co-leaders. In 2025, four 7th grade classrooms participated (n = 75 students), with 5 college leaders but no middle school student co-leaders. We stopped the youth leadership program in 2025 due to budget cuts and loss of staff members who had close relationships with students on the youth team.

The *Body Justice* lessons were delivered by 1-2 college facilitators who received eight hours of training. Training took place across two days (4 hour sessions) and was largely experiential, with role-play practice delivering the lessons and feedback about content delivery, leadership skills, managing the classroom, and handling difficult scenarios. In the years with youth co-facilitators, middle school student leaders were selected by school staff and trained over four one-hour meetings by school staff and college leaders by walking through activities and practicing leadership skills. Middle school student co-leaders had a range of co-facilitation duties, including managing materials and slideshows, sharing lived experience with the class, and summarizing student responses and feedback.

A within-participants evaluation assessed the curriculum’s impact over time. Baseline surveys were collected by teachers the week before the curriculum started. Post-curriculum surveys were collected on the last day of lessons (~5 weeks after baseline) by the classroom teacher and college leaders (*M* days from pre-survey = 34.48, *SD* = 4.10 days). Follow-up surveys were collected two months after the program/three months from baseline (*M* days from post-survey = 78.86; *SD* = 15.52 days). College leaders returned to classrooms to collect follow-up surveys alongside teachers and to present preliminary impact data to classrooms (a short presentation of who the program reached that year). All research activities were approved by the university’s institutional human research review board. Passive parental consent was obtained for research activities, where parents received a letter home and could opt out of lessons, data collection, or both.

### Body justice project curriculum

The *Body Justice Project* lessons are divided into four topics, each containing two 45-minute lessons (eight lessons total across six hours). Each lesson includes two active exercises to engage with the topic (dissonance-based or psychoeducational activities with media literacy components). Materials for the curriculum include a detailed leader guide with scripted sections and activity outlines, slideshows to accompany the lessons, student handouts with activities, and other materials for activities (e.g., post-it notes and large posters, copies of brand advertisements to analyze).

- **Cultural appearance ideals**: Students identify and critique the narrow cultural norms about attractive bodies, particularly how they exclude diverse identities. A flexible and individual definition of health is introduced.
- **Diet culture and non-diet nutrition**: Students learn about size-inclusive health and principles of intuitive eating, deconstruct health myths and good vs. bad foods, and reflect on ways that food represents culture.
- **Social media and appearance pressure**: Students discuss the impact of mass media and social media on appearance pressure, critiquing advertisements and redesigning their social media to promote diverse body images and messages.
- **Body autonomy**: Students learn body compassion and self-care, boundary setting, and how to value diverse bodies other than their own.

### Measures

Measures were selected to assess disordered eating, empirically-identified eating disorders risk factors, and to reflect evaluation priorities of school partners.

**Socio-demographic questions.** In their baseline survey, students reported their age, race and ethnicity (following recommendations from Kambanis and colleagues (22), gender identity and sexual identity (following 2022 recommendations from the *National Academies of Science, Engineering, and Medicine (*23*)*,, and languages spoken in their home (open response). Students also reported on current financial strain (24) and daily hours of social media use (25–27).

**Unhealthy Weight Control Behaviors.** Use of nine unhealthy weight control behaviors were measured at baseline and two-month follow-up, based on items from Neumark-Sztainer’s project EAT surveys with the time frame modified from 12 months to 3 months (28). Students responded yes no to whether they had engaged in the following behaviors to change their weight or shape: fasting, eating very little food, taking diet pills, vomiting, laxatives, diuretics, use of food substitutes, skipping meals, or following high protein or low carbohydrate diet. The nine items were used as a dichotomous outcome (use of zero unhealthy weight control behaviors or use of 1+ behavior). Four extreme weight control behaviors formed a separate dichotomous outcome (fasting, eating very little food, taking diet pills, and vomiting). Given the time frame for responses, weight control behaviors were not assessed post-program.

**Internalization of appearance ideals.** At pre-intervention, post-intervention, and two-month follow-up, investment in socially normative thin/lean and muscular appearance ideals was assessed with the 10 internalization items from the Sociocultural Attitudes Toward Appearance-Questionnaire 4 Revised (SATAQ-4R (29);. Scores were averaged across all 10 items, with higher scores indicating greater appearance ideal internalization. Our team commonly uses these subscale items as a single measure of appearance ideal internalization in college samples across the gender spectrum (18, 19). The entire SATAQ-4R was normed on a sample of early adolescent girls (ages 10-14) and it has been used in mixed gender adolescent samples (30). In the current sample, the 10 SATAQ-4R internalization items had very high reliability (Chronbach’s alpha = 0.88).

**Appearance Pressure from Social Media.** At pre-intervention, post-intervention, and two-month follow-up, appearance pressure from social media was assessed with the media questions from the SATAQ-4R (29). Items were adapted to address social media specifically (e.g., “I feel pressure from social media to improve my appearance.”) There was an accidental omission of one item (“I feel pressure from social media to look more muscular.”) The four remaining items were averaged to create a single score, with higher scores indicating greater appearance pressure from social media. In the current sample, the four social media pressure items had very high reliability (Chronbach’s alpha = 0.93).

**Body Justice satisfaction and application.** Immediately following the *Body Justice* lessons, students reported the number of sessions they attended and shared whether they enjoyed the lessons, understood them, thought the activities were fun, thought their leaders did a good job, and whether they would recommend the program to a friend. These last five items were averaged to create an overall satisfaction score (rated from 1-5 with higher scores indicating greater satisfaction).

At two-month follow-up, students reported key lessons/takeaways though an open-ended question meant to prompt their memory about intervention content. Students then responded to three frequency items rated from 1-5 about whether they still thought about the *Body Justice* lessons, whether they had made changes to their life based on what they learned, and whether they talked about the information with others. These items were averaged to create an overall application score, with higher scores indicating greater application of the *Body Justice* lessons at follow-up. Satisfaction and application questions are based on our team’s prior research with body image interventions in young adults (18, 19).

**Leader adherence and competence.** In order to assess leader adherence to the scripted curriculum and leader competence in facilitating the lessons, two sessions of each class were observed by an independent rater on the university research team. Only collegiate leaders were assessed on adherence and competence; youth leaders took on variable leadership roles (e.g. advancing lesson slides and helping facilitate activities) and as such were included in ratings of adherence. An adherence checklist was created for this research and asked raters to note whether key intervention elements (4-6 per lesson) were covered or not covered; this created an adherence rating across all eight lessons that ranged from 0-100%. This replicates a process used in other dissonance-based intervention research to assess facilitator adherence (19). Competence was rated with three items created for this research, all rated from 1-10 with higher scores indicating greater leader competency (1): overall impression of the session (broadest impression of how the content was delivered and received) (2), leader organization and time management (e.g., covering all content, giving time for student engagement), and (3) leader communication of acceptance and respect (e.g., through leader warmth, validation, and enthusiasm). Anchors were provided for even numbers along the 1-10 rating scale to enhance the consistency of competency ratings (2=room for growth, 4=below average, 6=average, 8=above average, 10=awesome).

### Analysis

Analyses were run using the Statistical Package for the Social Sciences (SPSS) version 31. Outcome data were screened for fit with the assumptions of planned statistical tests. For continuous outcomes, nonnormal continuous variables were transformed for analyses and baseline differences across social identity categories were examined with one-way Analysis of Variance (ANOVA).

To correct for a nonnormal distribution, SATAQ-4R internalization scores were transformed with Log10 for analyses; descriptive statistics reported are on untransformed data to ease interpretation. There were baseline differences in internalized appearance norms based on gender (*p* = .007), where boys reported higher internalization scores compared to girls and gender diverse students. There were no baseline group differences based on race and sexual identity. There were baseline differences in appearance pressure from social media based on gender (*p* = .008) where girls and gender diverse students reported higher appearance pressure compared to boys. There were also differences based on race (*p* = .009) where students of color reported higher appearance pressure compared to white students. There were no baseline differences based on sexual identity.

There was a significant amount of missing data across the sample (17% at baseline, 27% at post-intervention, and 24% at follow-up). In terms of adequate data for longitudinal analysis, 63% of students had both pre- and post-intervention data, 63% had both pre- and follow-up data, and only 53% completed their survey at all three time points. Little’s test indicated that survey responses were missing completely at random (MCAR; *p* = .199).

Change in categorical outcomes was examined with nonparametric tests (McNemar Change Test for related samples, Logistic Regression to include covariate predictors above and beyond time). Models were first run with the entire sample and then the following binary covariates were added to examine their contribution to outcome changes: gender, race, and sexuality. To preserve power in analyses, social identities were split into binary categories to create roughly equal groups: gender (cis male versus cis women and gender diverse students); race (split into white students vs. students of color); and sexuality (split into straight vs. sexually diverse).

In order to maximize the use of available data, multilevel Linear Mixed Models evaluated change in continuous outcomes over time. These models utilize full maximum likelihood estimation to account for missing data (31). Level 1 of mixed models included repeated measurement of the outcome nested within participants. Classroom and intervention year were initially included in models as a Level 2 covariate to adjust for nonindependence, but they were removed as they were not significantly related to outcome. Time was modeled at Level 2 as a non-linear effect. Model fitting and theoretical fit led to a First Order Autoregressive covariance structure for Level 1 and Diagonal covariance structure for Level 2. Linear mixed models were first run with the entire sample and then examined separately for social identity categories.

## Results

### Participant demographics

The average student age across the entire sample (of *N* = 234 students reporting demographic information) was 12.44 years, *SD* = 0.51. The sample was 51% white and 49% students of color (4% Black, 8% Asian, 5% Native American, 2% Hawaiian or Pacific Islander, 9% Multiracial, 2% another race, and 19% Latinx/e (any race). Students’ self-reported gender identity was 53% cisgender boys, 36% cisgender girls, and 12% gender diverse students (2% trans masculine, 4% non-binary, 5% gender questioning, and 1% another gender). Students’ self-reported sexual identity was 73% straight and 27% sexually diverse (6% lesbian or gay, 8% bisexual or pansexual, 3% asexual, 8% not sure, 1% questioning, and 1% another sexuality). **Table 2** reports all detailed demographic information across participants. A majority of students (59%) indicated that they spoke only English at home, with 12% reporting they spoke only Spanish and 29% reporting their family spoke multiple languages at home. A majority of students reported that their family had no financial insecurity (62%), with 38% reporting that their family had at least some financial insecurity (indicated as responding with a 1 or 2 on a 4-point scale). Students reported using social media an average of 3.08 hours/day (*SD* = 3.13, range 0-19 hours), and 20% of the sample reported that they spent zero hours per day on social media (suggesting they may not yet have access).

**Table 2:** Socio-demographic information for the total sample (N=234 reported).

Age (mean, SD, range)	12.44 (0.51), 11-13
Daily social media use (mean, SD, range)	3.08 (3.13), 0-19
Financial Insecurity (%) None Some	62% 38%
Languages spoken at home (%) English only Spanish only Multiple languages	60% 12% 29%
Racial identity (%) Black Asian White Native American Hawaiian or Pacific Islander Multiracial Another Race Latinx (any race)	4% 8% 51% 5% 2% 9% 2% 19%
Gender identity (%) Cisgender boy Cisgender girl Trans masculine Trans feminine Non-binary Gender questioning Another gender	53% 36% 2% 0% 4% 5% <1%
Sexual identity (%) Lesbian or gay Straight Bisexual or Pansexual Asexual Not Sure Questioning Another sexuality	6% 73% 8% 3% 8% 1% 1%

### *Body Justice* leader demographics

Of the 25 college leaders who self-reported demographic info (9 from 2023, 11 from 2024, 5 from 2025), around half were White (52%), 4% Black, 20% Asian or Asian-American, 20% Latinx (any race), and 4% Multi-racial. Self-reported sexual identity was 8% Gay or Lesbian, 32% Straight, 26% Bisexual or Pansexual, 8% Asexual, and 16% Another Sexual Identity. Leader gender identity was 12% Cis Men, 64% Cis Women, 8% Trans Masculine, and 16% Gender Non-Conforming.

### Intervention satisfaction and application

The majority of students attended either all eight *Body Justice* lessons (46%) or 6-7 lessons (43%). Across all students, 39% reported they did not have a favorite week of the *Body Justice* curriculum; a smaller proportion said that their favorite week of content was related to diet culture and non-diet nutrition (24%) or media and appearance pressures (22%). A small number stated that their favorite week was cultural appearance ideals (8%) or body autonomy (7%).

On average, students were moderately satisfied with the *Body Justice* curriculum (*M* satisfaction rating = 3.44, *SD* = 0.85). The highest endorsed item was “my leaders did a good job” (*M* = 4.06, *SD* = 0.93). There were differences in satisfaction with the curriculum based on social identity category. Students of color reported significantly higher satisfaction overall with the intervention (*M* = 3.72, SD = 0.84) compared to white students (*M* = 3.46, *SD* = 0.71; *p* <.001). Similarly, sexually diverse students reported higher satisfaction (*M* = 3.84, SD = 0.81) compared to straight students (*M* = 3.50, *SD* = 0.76; *p* <.001). Cisgender girls and gender diverse students reported greater satisfaction with the intervention (*M* = 3.79, *SD* = 0.70) compared to cisgender boys’ satisfaction (*M* = 3.36, *SD* = 0.84; *p* <.001).

At the two-month follow-up, students reported relatively low levels of application of the curriculum in general (*M* = 1.58 out of 5, *SD* = 0.65). Just 20% of students reported that they had talked to someone about what they learned in the program in the past two months. There were no differences in application based on students’ race (*p* = .490), but sexually diverse students reported greater application of *Body Justice* content at follow-up (*M* = 1.80, *SD* = 0.72) compared to straight students (*M* = 1.50, *SD* = 0.56; *p* = .015). Similarly, cisgender girls and gender diverse students reported greater application of new content in the follow-up period (*M* = 1.78, *SD* = 0.68) compared to cisgender boys (*M* = 1.44, *SD* = 0.59; *p* <.001). **Table 3** reports *Body Justice* satisfaction and application scores across the whole sample and by social identity category.

**Table 3:** Satisfaction and application scores for total sample and by social identity group.

Group	Satisfaction M (SD)	Application M (SD)
Total Sample	3.44 (0.85)	1.58 (0.65)
Students of Color White students	3.72 (0.84) 3.46 (0.71)	1.64 (0.66) 1.57 (0.64)
Cisgender boys Cisgender girls and gender diverse	3.36 (0.84) 3.79 (0.70)	1.44 (0.59) 1.78 (0.68)
Sexually diverse students Straight students	3.84 (0.81) 3.50 (0.76)	1.80 (0.72) 1.50 (0.56)

Satisfaction with the Body Justice Project was assessed with five questions post-intervention; Application of the Body Justice content was assessed with three questions at two month follow-up.

### *Body Justice* leader adherence and competence

Across the years of the *Body Justice Project*, leader adherence to the lesson plans ranged from 67%-100% in year 1 (*M* = 93%, *SD* = 8%), 63%-100% in year 2 (*M* = 85%, *SD* = 14%), and 80-100% in year 3 (*M* = 98%, *SD* = 7%). Overall facilitator competence was rated highly across years (*Ms* = 8.2 in year 1, 8.71 in year 2, and 8.50 in year 3). Facilitator organization was also rated highly and increased slightly over the years (*M*s = 7.90 in year 1, 8.07 in year 2, and 8.75 in year 3). Facilitator respect was the highest rated item across all years (*M*s = 8.90 in year 1, 9.07 in year 2, and 9.00 in year 3).

### Change in unhealthy weight control behaviors

**Figure 1:**
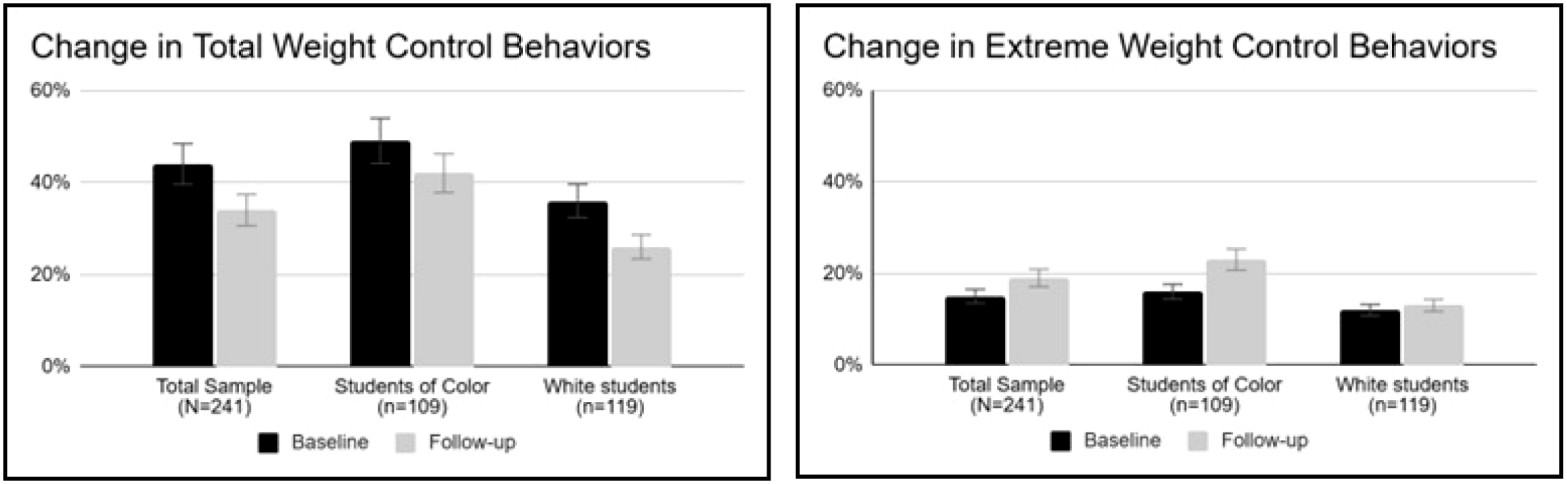
Change in unhealthy weight control behaviors across sample and by racial category.

Across the sample, there was a significant reduction in unhealthy weight control behaviors from baseline to follow-up (*p* = .007). At baseline, 44% of students reported one or more weight control behavior; this dropped to 34% at follow-up. When *extreme* weight control behaviors were examined, the change from pre-intervention to follow-up was not significant (*p* = .064; 15% of students pre-intervention and 19% at follow-up). Gender (*ps* >.448) and sexual identity (*p*s >.063) were not significant in predicting change in either total behaviors or extreme weight control behaviors. While race was not a significant predictor for change in *extreme* weight control behaviors (*p* = .449), it did significantly relate to total unhealthy weight control behaviors (*p* = .033). While reductions in unhealthy weight control behaviors were present across racial categories, more students of color reported unhealthy weight control behaviors at baseline (49%) and follow-up (42%), and changes over time were smaller relative to their white counterparts (36% at baseline, 26% at follow-up). **Figure 1** presents changes in unhealthy weight control behaviors changes over time across the whole sample and by racial category.

### Change in internalized appearance norms

Across the whole sample, there was a significant reduction in internalized appearance norms over time (*p* = .006) with a small effect size through follow-up (Cohen’s *d* effect size from baseline to follow-up = -0.11). When subgroups were examined based on social identity category, reductions over time were present for cisgender boys (*p* = .025, *d* = -0.29 at follow-up) and for girls and gender diverse students (*p* = .007, *d* = -0.17 at follow-up). Reductions in internalized appearance norms were present for white students (*p* = .007, *d* = -0.29 at follow-up) but not students of color (*p* = .179, *d* = -.05 at follow-up) and for straight students (*p* = .011, *d* = -0.24 at follow-up) but not sexually diverse students (*p* = .234, *d* = -.02 at follow-up). **Table 4** includes total sample and subgroup means, standard deviations and Cohen’s *d* effect sizes on magnitude of change from baseline to post-intervention and from baseline to follow-up.

**Table 4:** Appearance ideal internalization for total sample and by social identity group.

Group	Baseline	Post- intervention	Pre-post effect size	Two month follow-up	Pre-follow-up effect size
	M (SD)	M (SD)	*d*	M (SD)	*d*
Total Sample	2.63 (0.90)	2.61 (0.86)	-0.02	2.53 (1.00)	-0.11
Students of Color	2.69 (0.88)	2.71 (0.84)	0.02	2.65 (1.00)	-0.05
White students	2.58 (0.92)	2.39 (0.84)	-0.21	2.31 (0.95)	-0.29
Cisgender boys	2.79 (0.89)	2.51 (0.89)	-0.32	2.53 (1.05)	-0.29
Cisgender girls and gender diverse	2.47 (0.88)	2.53 (0.78)	0.07	2.32 (0.88)	-0.17
Sexually diverse students	2.42 (0.90)	2.47 (0.92)	0.06	2.40 (0.96)	-0.02
Straight students	2.73 (0.87)	2.57 (0.83)	-0.18	2.52 (1.02)	-0.24

Cohen’s *d* effect sizes using baseline standard deviation and a correlation between measures of 0.5.

### Change in appearance pressure from social media

Across the entire sample, there was no effect of time on change in appearance pressure (*p* = 0.324). However, when changes were examined within social identity categories, there was a significant reduction in appearance pressure for students of color (*p* = .039, *d* = -0.18 at follow-up) but not white students (*p* = .942, *d* = -0.05 at follow-up). There was no difference in change in appearance pressure based on gender (*p*s >.269) or sexual identity (*p*s >.714). **Table 5** includes total sample and subgroup means, standard deviations and Cohen’s *d* effect sizes on magnitude of change from baseline to post-intervention and from baseline to follow-up.

**Table 5:** Social media pressure for total sample and by social identity group.

Group	Baseline	Post- intervention	Pre-post effect size	Two month follow-up	Pre-follow-up effect size
	M (SD)	M (SD)	*d*	M (SD)	*d*
Total Sample	2.24 (1.19)	2.27 (1.60)	0.03	2.14 (1.18)	-0.08
Students of Color	2.49 (1.18)	2.60 (1.08)	0.09	2.28 (1.13)	-0.18
White Students	2.01 (1.14)	2.03 (1.15)	0.02	1.95 (1.13)	-0.05
Cisgender boys	2.04 (1.08)	2.04 (1.07)	0.00	1.87 (1.11)	-0.16
Cisgender girls and gender diverse	2.45 (1.25)	2.61 (1.21)	0.13	2.39 (1.13)	-0.05
Sexually diverse students	2.24 (1.27)	2.40 (1.33)	0.13	2.27 (1.32)	0.02
Straight students	2.24 (1.15)	2.32 (1.12)	0.07	2.12 (1.13)	-0.10

Cohen’s *d* effect sizes using baseline standard deviation and a correlation between measures of 0.5.

## Discussion

This implementation-effectiveness study examined the impact of a justice-focused, universal prevention intervention for middle school students across three years of implementation using near peer college leaders in 7th grade classrooms. The body image curriculum and evaluation plan was co-created in a participatory research partnership between university researchers and students and staff within a local middle school. In general, students who received the *Body Justice* lessons were moderately satisfied with the curriculum but reported low levels of application of new information from the curriculum at follow-up. *Body Justice* leaders had high adherence to the intervention manual and were rated as highly competent, suggesting that this is a feasible intervention. Overall, we found that the *Body Justice* lessons had a positive impact on students, though the impact may not be equal across students in different social identity subgroups, with important implications for culturally-based mental health promotion. It’s worth noting that the findings across subgroups should be interpreted with some caution given our limited power and category aggregation.

Across all students who participated in *Body Justice* lessons, there was a significant improvement in unhealthy weight control behaviors over time. Nearly half of the sample (44%) reported one or more unhealthy weight control behavior at baseline, rates that are similar to those found in school-based longitudinal research with racially and ethnically diverse adolescents (28). This suggests that students who received the *Body Justice* lessons represented a typical sample for universal prevention efforts. Rates of unhealthy weight control behaviors dropped by 10% at two-month follow-up, which is a small but encouraging decrease. Given that effects of interventions attenuate over time, a modest reduction in behavior is impactful to see two months after a universal prevention intervention. Research shows that eating disorder symptoms that emerge in late adolescence tend to be stable or worsen over time without intervention (8), suggesting that reductions in unhealthy weight control behaviors in early adolescence may be meaningful in changing the trajectory of eating disorder risk over time.

When *extreme* weight control behaviors were examined (the presence of fasting, eating very little food, taking diet pills, and vomiting), there was no significant improvement over time; this is not surprising given that the *Body Justice* program was designed as prevention and not meant to target students with current eating disorder symptoms. However, it is notable that the extreme weight control behaviors reported by students in our sample are much higher than rates published in previous research (11.3% of cisgender boys in our sample compared to 3.9% in a 2010 Project Eat sample; 15.9% of cisgender girls and gender diverse students in our sample compared to 6.8% in a 2010 Project EAT sample) (28). Although unhealthy weight control behaviors are not a diagnostic measure of eating disorders, this may reflect the rising incidence of eating disorders globally (3) and warrants future research to assess ways to screen and refer early adolescents within schools to connect them to appropriate mental health services.

Across the sample, there also was a statistically significant decrease in students’ investment in cultural appearance ideals over time (through post-intervention and two-month follow-up), which is a key eating disorder risk factor and a theoretical target of the dissonance-based components of the *Body Justice* intervention (5). The effect size for this change across the entire sample was quite small, but even a small change in a low-risk, universal population can be impactful (32), particularly at a point in adolescent development that precedes the onset of disordered eating and eating disorders (8). Interestingly, there was no change in the appearance pressure students reported from social media over time. Comparisons of baseline scores for both of these outcomes, measured with the SATAQ-4R, shows that while internalization scores are similar to those found in adolescent community samples, scores on appearance pressure were somewhat lower in the current sample (29). It is possible that a floor effect limited our ability to see change in this outcome. It is also possible that the two lessons on media (out of eight lessons total) were not sufficient to target this risk factor. Social media use (especially visually-based social media like Instagram, Tiktok, etc.) has been established as an important contributor to appearance-based comparison, body dissatisfaction, and eating disorder symptoms among adolescents (33), and future research should target this area of prevention work. The adolescents in our sample were around 13 years old; it is possible that they do not have access (or full access) to social media yet. Students shared that they spent an average of 3.08 hours per day on social media (which is slightly lower than national norms (34), and 20% of the sample reported spending zero hours per day on social media. Future research should continue to examine how to best target appearance pressure from social media through body image programming. For example, interventions could include more interactive activities that involve real-time evaluation of algorithm driven content exposure. This would further personalize the lessons and allow participants to critically analyze appearance ideals on their own social media feeds.

Another aim of this research was to create an intervention to specifically address the body image concerns of culturally diverse adolescents with identities that are marginalized based on appearance and underrepresented in body image programming and research. Given the relatively high representation of students of color (49%) and sexually diverse students (27%) in our sample, we were able to analyze effects of the *Body Justice* lessons by these identity category subgroups compared to their majority-identity peers (white students and straight students, respectively). We also examined the impact of the curriculum for cisgender boys (53%) compared to a combined group of cisgender girls and gender diverse students. Notable patterns emerged from sub-group analyses, which could suggest that the impact of *Body Justice* programming was not equal across all students, though these patterns should be interpreted cautiously given the large amount of missing data within the sample. We found higher satisfaction with the *Body Justice* curriculum among students with marginalized identities compared to their majority peers, as well as greater application of *Body Justice* material for cisgender girls and gender diverse students and sexually diverse students compared to their majority peers, though not for students of color. This suggests that the content of this curriculum may reflect the priorities and experiences of students with marginalized identities around their typical body image concerns. When possible, we also tried to match college student leadership to the diversity within the school setting, and this may have resulted in increased satisfaction and application of the intervention content.

However, when outcome data were examined by these social identity groups, the patterns may suggest differential impacts for students with marginalized identities. In general, students of color were at higher risk for developing an eating disorder at baseline relative to their white peers (endorsing greater baseline scores on appearance pressure from social media and a higher proportion endorsing unhealthy weight control behaviors). While students in both racial groups reduced their unhealthy weight control behaviors over time, the reductions were slightly smaller among students of color (7%) compared to white students (10%). Furthermore, students of color did not experience a reduction in internalization over time while their white peers did, but students of color *did* experience significant improvements in appearance pressure from social media while their white peers did not. In fact, students of color were the only sub-group who experienced any significant change in appearance pressure from social media during the *Body Justice* curriculum.

It is somewhat difficult to parse this pattern of findings around students’ racial identity and their experience with the *Body Justice* intervention. There were attempts to represent the experiences of students of color by incorporating racially diverse images where applicable (e.g., discussing body ideals) and explicitly naming cultural food considerations and traditions (e.g., through the favorite food memory activity). Students on the youth advisory team, staff within the school, and researchers at the university – who were responsible for creating the curriculum and planning the research – had a variety of diverse and intersecting identities. Yet, it is possible that appearance norms did not feel as relevant to students of color in this sample, and therefore they may have been less susceptible to change during *Body Justice* lessons. Research on eating disorder risk among communities of color in the United States has been mixed, with some research finding that risk factors normed on white populations such as appearance ideal internalization do not translate to risk among populations of color, where body ideals may vary based on racial and ethnic identity (e.g., a curvier feminine body ideal in Black and Latine communities compared to the thin White ideal) in ways that are protective rather than harmful (35). However, exposure to dominant white body ideals are difficult to avoid, and future research should examine how awareness and internalization of the idealized white body represented in the media contributes to body dissatisfaction and disordered eating among individuals of any race (35). Prior research on disordered eating patterns is clearer, finding that rates of disordered eating among adolescents of color are similar to their white counterparts and at times, even higher (e.g (35, 36). As such, future research may benefit from shaping an intervention to students of color as much as possible (including guiding theoretical frameworks) to try and accurately capture the experiences of these student populations.

In addition to sociocultural influences, contributors to disordered eating and eating disorder symptoms among people of color includes discrimination and acculturative stress (37). Because the intervention was conducted in a predominantly white town, body and image standards may also be shaped by the norms and/or stressors in the local community. That is, it is possible that the demographics of the location could be contributing to the initial endorsement of weight control behaviors and maintaining internalized beliefs around ideal body image expectations. With the change we saw in relation to social media for students of color, it may be that social media is a space to view and/or engage with people who reflect their racial/ethnic identities as a matter of further developing a racialized sense of self. Given the elevated risk of students of color in the current study, it is promising that they did show improvement in some outcomes, though future research should continue to adapt interventions to meet the specific needs of racial groups at highest risk.

Outcome sub-analyses based on gender followed different patterns. While cisgender boys had higher baseline scores around internalization of appearance ideals, they experienced a steady improvement in scores over time. Cisgender girls and gender diverse students had an initial worsening of internalization scores post-intervention, but they experienced reductions similar to cisgender boys by the two-month follow-up. This fluctuation could be explained by pre-intervention attitudes and comparison to others. That is, cisgender boys could only be comparing themselves to other boys (peers) with more limited external comparisons, and the novelty of information related to body image could have led to a greater impact on this outcome. Cisgender girls, however, may be comparing themselves to other girls plus external media. Another possible explanation is that cisgender girls may be internalizing more factors related to appearance (e.g., physique, clothes, hair) than cisgender boys, which could also be similar for gender diverse students. Gender did not significantly contribute to change in unhealthy weight control behaviors over time (improvements were present across gender categories), and while cisgender girls and gender diverse students reported greater appearance pressure from social media at baseline, there was no significant change over time across the whole sample, regardless of gender.

There is ample research demonstrating that cisgender girls and young women are at elevated risk for body image concerns and eating disorders compared to cisgender boys (e.g (2, 3); recent research also documents the uniquely elevated risk of gender diverse young people (e.g (38). Interestingly, some prior research finds that school-based prevention programs are generally less effective for girls compared to boys (39), though we did not find this effect. Recent research is also beginning to explore how intersecting marginalized identities can impact eating disorder risk among adolescents, and future studies should aim to consider how the combination of gender *and* another marginalized identity may impact risk. Similar to the risk factor work within communities of color, prevailing theories suggest that stigma and discrimination contribute to body image concerns and disordered eating among gender diverse individuals (38).

In general, sexual identity did not impact the outcomes of the *Body Justice* intervention, with equal impact on change in unhealthy weight control behaviors and no impact on appearance pressure from social media. However, straight students reported improvement in internalized appearance ideals, while sexually diverse students did not. The proportion of students who were sexually diverse in the current sample was relatively small (just 27% of students), and it is possible that these sub-analyses were particularly underpowered to examine differences based on this identity category, if they do exist. Existing research has documented that sexually diverse adolescents report disproportionately high rates of disordered eating (40), and so it is encouraging that some aspects of the *Body Justice* intervention had a positive impact on students with diverse sexual identities.

### Strengths and limitations

Strengths of this study include having a racially and gender diverse sample and multiple assessment points over time. The community-engaged research methods of this work are a strength, as they allowed us to co-create an intervention with student voices at the center, as well as caring adults within the school environment. However, the intervention and results of this research may not be replicable in other contexts. While efforts were made to culturally adapt the intervention and intentionally incorporate more information pertaining to the experiences of marginalized students, the muted impact on some outcomes suggests there is room for improvement. It is important that the development of future/similar interventions, and subsequent iterations, center the experiences of marginalized students in order to ensure that their experiences are not further marginalized nor that they feel the material is not as applicable to their experiences.

Another limitation was not having a comparison group for this intervention. Without an experimental design and control group we cannot be certain that any changes we see are not due to the passing of time or other confounds that we did not control for. We also were unable to explore whether specific content or group makeup (e.g., having students with similar marginalized identities in affinity groups together) impacted outcomes or the experience of the intervention. Social desirability may impact reporting of sensitive topics like disordered eating, making it difficult to accurately assess this information. This design was not possible for this research collaboration, as the school wished for all students to receive the curriculum on the same timeline each year. Other limitations include the high amount of missing data within the sample (63% of students had both pre- and post-intervention data; 63% had both pre- and follow-up data, and only 53% completed their survey at all time points). While our use of mixed models and testing for missingness (i.e., MCAR analysis) helped account for the missing data within the sample, attrition may have still influenced the longitudinal findings. Though combining samples across multiple years of implementation allowed us to still do sub-group analyses and explore impacts based on broad identity categories and multi-level analyses allowed us to model changeover time with as few data points as possible, these analyses are still underpowered to detect interaction effects that may be present. Additionally, combining some groups (e.g., cisgender girls and gender diverse students) may have masked unique effects across these groups that would warrant more attention. Future research should aim to explore racial, sexual, and gender identity differences in finer detail to help better understand how interventions impact specific identity-based experiences and their intersections.

## Conclusions

This research demonstrates the potential of a culturally-tailored, evidence-informed body image intervention for early adolescents that was co-created by middle school students and adults invested in their well-being. Our findings suggest there could be differential impacts of the curriculum based on social identity categories such as race, gender, and sexuality, and we encourage future prevention research to continue exploring ways to adapt programming to meet the specific needs of diverse and intersecting identities. While co-creating the intervention and evaluation plan with youth and adult advisory teams was both intentional and effortful, we encourage research teams to include the perspectives of lived experience when creating programming for young people. While we were not able to include the perspectives and support from families in this research, it is also important to involve caregivers and other support systems within prevention work in order to educate the whole social context around body liberation.

We also encourage continued universal prevention work with this age group for two reasons. First, the burden of eating disorders will be lifted if we can intervene and support young people before their symptoms develop or as shortly thereafter as possible. Second, given the pervasiveness of body image concerns and their negative impact on overall mental health, interventions should feel comfortable addressing this construct as early and often as possible within settings like schools where all students can receive this education. While many school-based interventions utilize teachers to deliver interventions created by research teams, our co-design process revealed the strong desire to *not* involve teachers as facilitators, given the significant workload they already carry. Use of “near peers” in the form of trained college leaders was feasible and effective, and identifying novel facilitators for interventions may be a good implementation model for teams moving forward. Finally, given the focus on social identity categories within this research, we want to emphasize that social identities such as race, gender, and sexuality do not carry risk or resilience for eating disorders development *on their own;* rather, it is the experience of embodying an identity that is judged – and often discriminated against – based on appearance and its representation of these social categories, that creates the increase in vulnerability or resistance.

## Data Availability

The raw data supporting the conclusions of this article will be made available by the authors, without undue reservation.
